# *SAMD9* and *SAMD9L* in inherited predisposition to ataxia, pancytopenia, and myeloid malignancies

**DOI:** 10.1038/s41375-018-0074-4

**Published:** 2018-02-25

**Authors:** Josef Davidsson, Andreas Puschmann, Ulf Tedgård, David Bryder, Lars Nilsson, Jörg Cammenga

**Affiliations:** 1 Department of Pediatric Hematology and Oncology, Skåne University Hospital, Lund, Sweden; 2 Department of Molecular Hematology, Lund University, Lund, Sweden; 3 Skåne University Hospital, Department of Clinical Sciences Lund, Neurology, Lund University, Lund, Sweden; 4 Department of Hematology, Oncology and Radiation Physics, Skåne University Hospital, Lund, Sweden; 5 Department of Hematology, University Hospital Linköping, Linköping, Sweden; 6 Institution for Clinical and Experimental Medicine, Linköping University, Linköping, Sweden

**Keywords:** Cancer genetics, Genetics research

## Abstract

Germline mutations in the *SAMD9* and *SAMD9L* genes, located in tandem on chromosome 7, are associated with a clinical spectrum of disorders including the MIRAGE syndrome, ataxia–pancytopenia syndrome and myelodysplasia and leukemia syndrome with monosomy 7 syndrome. Germline gain-of-function mutations increase *SAMD9* or *SAMD9L*’s normal antiproliferative effect. This causes pancytopenia and generally restricted growth and/or specific organ hypoplasia in non-hematopoietic tissues. In blood cells, additional somatic aberrations that reverse the germline mutation’s effect, and give rise to the clonal expansion of cells with reduced or no antiproliferative effect of *SAMD9* or *SAMD9L* include complete or partial chromosome 7 loss or loss-of-function mutations in *SAMD9* or *SAMD9L*. Furthermore, the complete or partial loss of chromosome 7q may cause myelodysplastic syndrome in these patients. *SAMD9* mutations appear to associate with a more severe disease phenotype, including intrauterine growth restriction, developmental delay and hypoplasia of adrenal glands, testes, ovaries or thymus, and most reported patients died in infancy or early childhood due to infections, anemia and/or hemorrhages. *SAMD9L* mutations have been reported in a few families with balance problems and nystagmus due to cerebellar atrophy, and may lead to similar hematological disease as seen in *SAMD9* mutation carriers, from early childhood to adult years. We review the clinical features of these syndromes, discuss the underlying biology, and interpret the genetic findings in some of the affected family members. We provide expert-based recommendations regarding diagnosis, follow-up, and treatment of mutation carriers.

## Introduction

Aberrations of chromosome 7 in myeloid malignancies was first described in the 1960’s, but the exact gene(s) in this region driving myelodysplastic syndrome (MDS) and acute myeloid leukemia (AML) development remain elusive [1]. Besides total chromosomal loss (monosomy 7) or loss of the long arm of chromosome 7 (7q-), different commonly deleted regions (CDR) have been identified, including 7q22 (CDR1), 7q34 (CDR2), and 7q35-36 (CDR3) [2–4]. Interestingly, aberrations of chromosome 7 are found in 30–40% of pediatric MDS patients [5, 6], but only in 10% of adult MDS, for unknown reasons [7]. Despite decades of research striving to identify the gene(s) on 7q contributing to myeloid malignancies, very few mutations in the remaining haploinsufficient alleles have been found: in essence, only recurrent mutations in *CUX1* [4, 8] and *EZH2* [9] have been identified. This indicates that haploinsufficiency for genes located in the CDR1-3 is the principal mechanism driving MDS development in these cases.

For the myelodysplasia and leukemia syndrome with monosomy 7 (MLSM7; OMIM #252270), the presence of a sibling pair both diagnosed with a myeloid neoplasm displaying monosomy 7 is a prerequisite for diagnosis [10]. No candidate gene(s) has been ascertained for MLSM7 and the disease does not follow a clear monogenic pattern [11]. It has even been suggested that the predisposing locus is not located on chromosome 7 due to retainment of different parental chromosomes 7 between siblings with leukemic bone marrows [12].

The ataxia–pancytopenia (ATXPC) syndrome (OMIM #159550) was first described in 1978 by Dr. Frederik Li in a family with neurologic symptoms and pancytopenia of unknown origin, with some family members developing a myeloid neoplasm with monosomy 7 [13], Exclusion and linkage analyses combined with whole exome sequencing (WES) of an ATXPC kindred revealed mutations in the gene *SAMD9L* as the cause of this syndrome, and mutations in this gene were also found in the original family described by Dr. Li [15].

We were part of a collaborative team describing two families—from Sweden and Finland, respectively—with *SAMD9L* mutations and neurological symptoms and fluctuating cytopenias, infections, and MDS [16, 17]. In light of these recent findings, it is unlikely that ATXPC and MLSM7 really represent two distinct entities, but merely reflect a variation in the penetrance and severity of symptoms in one and the same disease spectrum that includes germ line mutations in e.g., *GATA2*, *SAMD9L,* and *SAMD9*, leading to monosomy 7. In this review, we summarize the disease phenotype in *SAMD9L* and *SAMD9* mutation carriers, and we attempt to provide recommendations for the identification, initial screening, follow-up and treatment of patients with, based on the limited data currently available about these disorders.

## SAMD9L

### Function of SAMD9L

SAMD9L, a protein widely expressed across human tissues, contains a SAM domain that binds RNA as well as oligomerizes both with SAM-containing and non-SAM-containing proteins. The exact role of *SAMD9L* is currently unknown, but the gene has a general antiproliferative function and has been demonstrated to function as tumor suppressor in breast, hepatocellular and in squamous cell carcinoma, being repressed by the p53 pathway [18]. In hematopoetic tissue *SAMD9L* regulates cell proliferation by being a crucial component of a protein complex that facilitates the degradation of cytokine receptors through the homotypic fusion of endosomes[19].

### Heterozygous germline mutations in SAMD9L in ATXPC syndrome

**Fig. 1:**
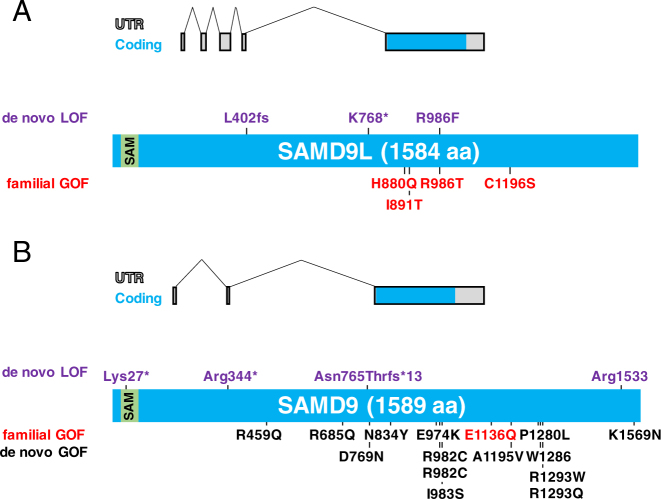
Disease-associated and somatic reversion mutations in *SAMD9L* and *SAMD9*. **a** Overview of the *SAMD9L* gene and protein (blue), including the SAM domain (green). Positions of all identified disease-associated germ line gain-of-function mutations (red) and somatic reversion loss-of-function mutations (lilac) reported to date are indicated. **b** Overview of the *SAMD9* gene and protein (blue), including the SAM domain (green). Positions of all identified disease-associated de novo (black) and germ line (red) gain-of-function mutations as well as somatic reversion loss-of-function mutations (lilac) reported to date are indicated

**Fig. 2:**
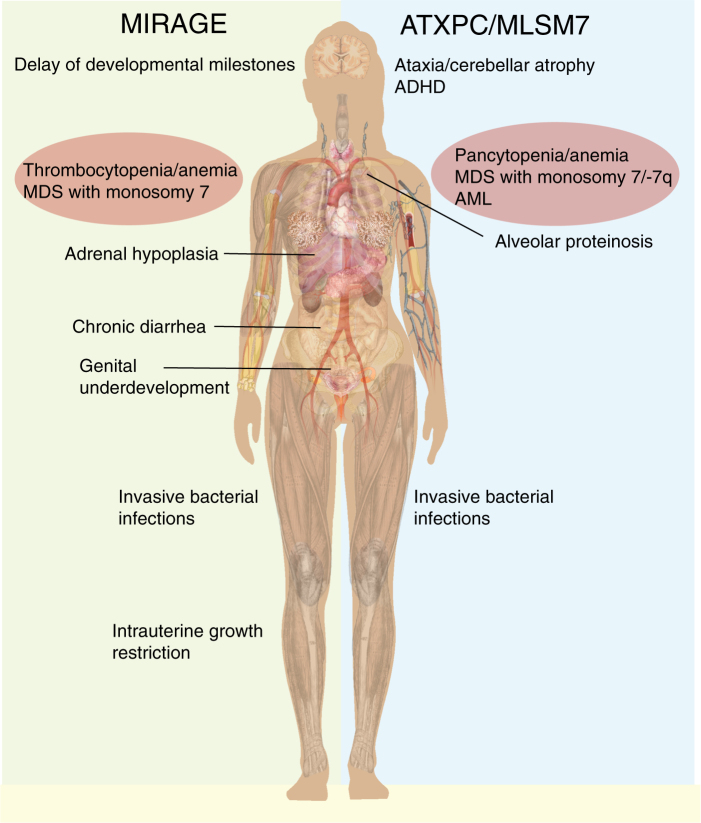
Known physical manifestations of the MIRAGE and ATXPC syndrome. The *SAMD9L* or *SAMD9* mutations’ antiproliferative effect may underly the general intrauterine growth reduction or organ-specific hypoplasias or atrophies observed in these syndromes. In *SAMD9L*-associated disease the non-hematological disease manifestations may be mild and patients may not report symptoms, but some degree of nervous system involvement was noted almost all ATXPC patients

To date, four families with the ATXPC syndrome and *SAMD9L* mutations have been described (Fig. 1a) [15–18]. In total, 25 mutation carriers have been identified. Eighteen had documented transient or permanent cytopenia, and eight of these had recurrent or severe infections or hemorrhages. Five individuals developed hematological malignancies at ages 1.5, 4, 7, 10, and 56 years. Except for one 4-year-old and one 16-year-old mutation carrier, all displayed either gait disturbance or nystagmus, or both (Table 1; Fig. 2). All mutations identified so far have been missense and none have been recurrent between families. The first observation led to the hypothesis by Chen et al. that germline mutations in *SAMD9L* result in a gain-of-function (GOF) [15], not a loss-of-function (LOF) and haploinsufficiency. In the latter theory, LOF/haploinsufficiency for *SAMD9L* in ATXPC would be the first hit towards leukemic transformation, followed by monosomy 7 or 7q- that includes other genes involved in the pathogenesis of MDS. This idea was however proven wrong by functional assays ascertaining that the germline mutations actually are GOF aberrations increasing the antiproliferative effect of *SAMD9L*[16].

**Table 1:** Summary of clinical phenotype of reported patients with ATXPC /MLSM7 and MIRAGE

Syndrome	ATXPC/MLSM7	MIRAGE
Gene	*SAMD9L*	*SAMD9*
Inheritance pattern	Autosomal dominant	Autosomal dominant, mainly *de novo* mutations
No. of families described to date	4	18
Hematological disease manifestations	Transient or permanent cytopenias with mild or no infections or hemorrhages; myeloid malignancies	Transient or permanent cytopenias with mild or no infections, or hemorrhages; myeloid malignancies
Age at onset	Infant–childhood–adult	Infant
Age at death	Adult	Infant–childhood
Non-hematological disease manifestations	Neurological (gait disturbance, nystagmus, cerebellar atrophy, pyramidal signs, cerebral white matter changes) Ophthalmological (retinal dysfunction) Behavioral abnormalities (in some patients, especially during childhood/adolescence) Mild to moderate facial, hand or feet dysmorphism (in some patients)	Intrauterine growth reduction Developmental delay Adrenal insufficiency, External genital abnormalities, Chronic enteropathy
Non-hematological pathology	Nerve cell loss (Purkinje cells, retinal cells)	Hypoplasia of adrenal glands, testes/ovaries, thymus
Proposed mechanism	Gain-of-function	Gain-of-function
Effect	Increased antiproliferative effect	Increased antiproliferative effect

### SAMD9L mutations are lost due to aberrations of chromosome 7 during progression towards MDS

The *SAMD9L* gene is located on chromosome 7q21.2 in the MDS CDR1 [19]. In the three patients identified to date with inherited GOF *SAMD9L* mutations who developed MDS, the mutant allele was lost due to aberrations of chromosome 7 [15, 16]. Importantly, a clear correlation between metaphases carrying the aberration of chromosome 7 and the change in the ratio between wild type (wt) and mutant *SAMD9L* ratio could be observed with disease progression [16]. One patient developed a der(1;7) which resulted in loss of genetic material on chromosome 7, including the mutant *SAMD9L* allele [16]. The other two patients that developed MDS lost the mutant *SAMD9L* copy by monosomy 7 and deletion of 7q, respectively [15, 16]. Again, we could clearly show a correlation between the metaphases carrying the aberration of chromosome 7 and the change in the variant allele frequency (VAF) towards the wt allele [16].

### Reversion of the germline SAMD9L mutation

Sequencing revealed a mutant *SAMD9L* VAF lower than the expected 50% in the blood in some of the unaffected family members. Furthermore, SNP array B-allele frequencies demonstrated clear skewing towards the wt allele, without net genomic loss in the region on chromosome 7q21.2. This implies that the low mutant VAF was due to a copy neutral loss of heterozygosity (CN-LOH) of chromosome 7q [16]. Chen et al. also observed decreased frequency of the missense mutant *SAMD9L* in two of their study subjects and suspected hematopoietic mosaicism. Long-term in vitro culturing of transformed lymphocytic lines from heterozygous carriers demonstrated that CN-LOH of 7q, resulting in net loss of mutant allele, occurred after 3 weeks to 6 months in culture. This is most likely due to the selective growth advantage attributed to hematopoietic precursor cells that have lost their mutated copy and duplicated the wt counterpart of *SAMD9L* by uniparental disomy (UPD) [15].

**Fig. 3:**
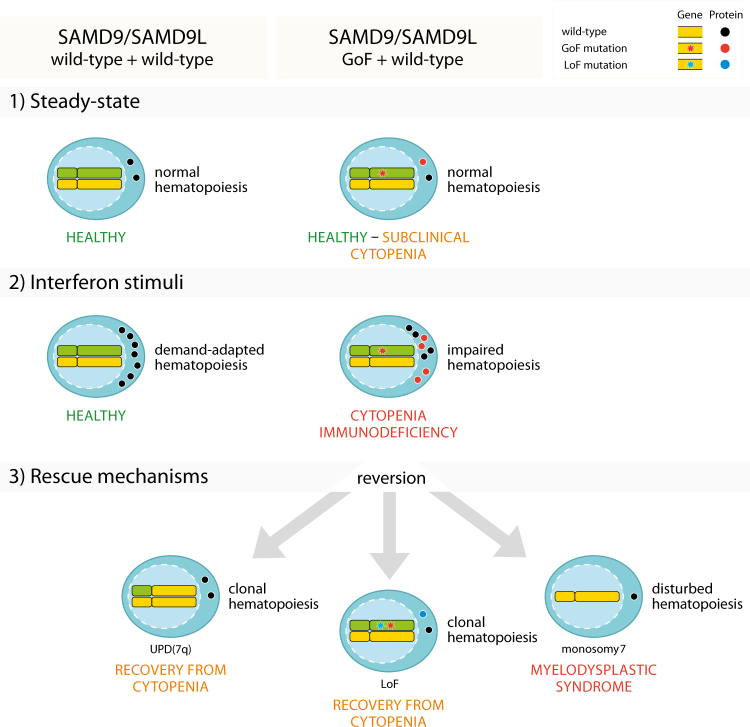
Overview of the genetic reversion mechanisms associated with gain-of-function *SAMD9*/*SAMD9L* mutations and their effect on hematopoiesis. Three different mechanisms of genetic reversion have been associated with gain-of-function mutations in the *SAMD9* and *SAMD9L* genes, often even in the same patient. Firstly, uniparental isodisomy by homologous recombination of the long arm of chromosome 7 results in replacement of the mutant allele with a wt copy and restores hematopoiesis. Secondly, somatic loss-of-function mutations *in cis* inactivates the GOF mutation and also restores hematopoiesis. Lastly, monosomy 7, del(7q) or der(1;7) all are “adaption by aneuploidy”, where the mutant allele is eliminated by total or partial loss of chromosome 7, on which SAMD9/SAMD9L are located, however, not restoring hematopoiesis but leading to MDS

Moreover, in some individuals the GOF mutation was retained but instead counteracted by frameshift/stop-gain mutations in *SAMD9L in cis* (Fig. 1 and Fig. 3) [16]. Normally frameshift/stop-gain mutations would result in destruction of the mRNA from the mutant allele by nonsense-mediated decay but this requires that the mutation occurs in a defined distance to intron/exon boundaries [20]. In the case of *SAMD9L*, functionally being a one exon gene, nonsense-mediated decay is thus likely not responsible for any LOF. As an additional mechanism of reversion, we identified *in cis* mutations in the same codon that carried the GOF mutation (Fig. 1a). These resulted in amino acid changes probably inducing less GOF, a hypothesis supported by proliferative effects seen in cells positive for *in cis* mutations when overexpressing the different *SAMD9L* variants in 293T cells [16].

Strikingly, some patients carried multiple clones with different genetic mechanisms of reversion and in one case a patient even carried a reversion by CN-LOH and additionally an MDS clone with aberrations of chromosome 7 [16]. These findings show the tremendous selection pressure of the cells to neutralize the GOF *SAMD9L* mutation and the advantage inferred to the cells that have undergone genetic reversion (Fig. 3). Family members that did not develop MDS all showed genetic reversions in some form or another, which is relevant in regard to the diagnostic criteria that at least two siblings have to be affected by MDS with aberration of chromosome 7 to make the diagnosis MLSM7. It appears likely that the common occurrence of genetic reversion has led to underdiagnosis of MLSM7. It can also be speculated, as in the study by Shannon et al. that investigated three families with MLSM7 and found retainment of mixed parental alleles in leukemic bone marrow between siblings, actually reflected mosaicism of clones harboring mutational reversal events and 7q-, respectively [12].

### The origin of SAMD9L mutations in hematopoietic hierarchy and development

*SAMD9L* is most highly expressed in NK cells and monocytes and subsequently symptomatic carriers usually present with low numbers of NK cells and monocytes [16]. To investigate the effect of the genetic reversion in mature cells; B-, T-, NK cells, monocytes, and granulocytes were isolated from two individuals affected by either CN-LOH or reversion mutations. Detection of allelic ratios of the wt and mutant *SAMD9L* showed that NK cells showed a higher percentage of gene reversion, indirectly arguing for the essential gene function of *SAMD9L* in NK-cell differentiation [16].

To investigate when the reversion of the inherited GOF *SAMD9L* mutations occurred, mutant VAF in peripheral blood (PB) at birth (using Guthrie cards) was investigated. Equal fractions of wt and mutant *SAMD9L* in PB directly after birth indicated that the low mutant *SAMD9L* VAF detected later in life was not caused by mosaicism at birth [16], but instead by a clonal genetic event that occurred most likely between 1 and 4 years of age. This also coincided with a time when many individuals were experiencing a transient pancytopenia, probably undergoing genetic reversion leading to a fitness advantage of the reverted cells, resulting in their expansion and recovery of the PB counts.

Cell sorting of hematopoietic stem cells (HSC), multi-potent progenitors (MPP), common myeloid progenitors, common lymphoid progenitors and granulocyte–monocyte progenitors followed by analysis of the mutant VAF using digital droplet PCR was performed to investigate at which cellular level the reversion has occurred. Surprisingly, this analysis suggested that the molecular reversion occurred at the MPP rather than at the HSC level [16], although more patients would have to be studied to draw more definitive and generalized conclusions. We hypothesize that the reversion of the inherited *SAMD9L* mutation might result in increased proliferation of MPPs (at least when compared to mutant cells), leading to clonal expansion. Alternatively, a rare HSC clone would represent the initial revertant, with the correction leading to a selective recruitment of these HSCs into differentiation but with no evident advantage in terms of self-renewal. In both cases, MPPs must associate with a high maintenance capacity. While most hematopoietic homeostasis has previously been attributed to HSCs, mainly based on transplantation experiments, such scenarios appear in line with more recent lineage-tracing studies and selective HSC-depletion strategies in mice, demonstrating substantial long-term contribution of MPPs not only in steady-state but also the context of stress hematopoiesis [21–24].

### SAMD9L knock-out mice develop MDS

It has been suggested that monosomy 7, 7q-, or 7q21 microdeletions increase the signaling through the cytokine cascade by the loss of *SAMD9* and *SAMD9L*, causing delays in homotypic endosome fusion in which these proteins partake, resulting in persistence of ligand-bound receptors [1]. To test this, a *Samd9l* knockout mouse was generated. Interestingly, both hetero- and homozygous *Samd9l* knockout mice developed an MDS-like phenotype with long latency. Of note, the latency was not significantly different between mice carrying one or two functional copies of the gene, arguing that haploinsufficiency alone might be sufficient for MDS development [19]. However, whether the other *Samd9l* allele was lost in heterozygous mice during transformation to MDS was unfortunately not investigated. Hence, it remains an open question as to what extent the *Samd9l* mouse model mimics the MDS phenotype observed in ATXPC patients.

Nagamachi et al. provided substantial evidence that SAMD9L is involved in endocytosis and receptor recycling affecting signaling through the *KIT* receptor [19]. This observation probably explains the advantage of *Samd9l*^−/−^ and *Samd9l*^+/−^ cells in the setting of a competitive transplantation, even though the advantage is rather small and is probably enhanced by the competition in the post-transplantation setting and/or by infections post transplantation that trigger interferon associated *Samd9l* expression. The observation that cells with reduced or absent levels of *SAMD9L* have a growth advantage compared to wt cells fits well with the observation that GOF *SAMD9L* mutations are leading to growth inhibition [16].

### Neurological phenotype of the ATXPC syndrome

Some degree of nervous system involvement has been documented in almost all carriers of pathogenic *SAMD9L* mutations reported to date (Fig. 2). Balance problems and nystagmus are most common, followed by mild pyramidal signs [17]. A few patients developed mild cerebellar-type dysarthria. Other cerebellar motor signs such as impairment of rapid alternating movements, dysmetria, cerebellar tremor were typically less pronounced than the balance problems, or entirely absent, why the usage of the term “ataxia” is actually debatable [17]. Moreover, we have identified paracentral retinal dysfunction in two mutation carriers and additional reported patients had clinical signs and symptoms highly suggestive of this. We therefore suggest that part of the dysmetria reported in the other families could be motor problems caused by limited visual control. Behavioral symptoms have been described in carriers of specific SADM9L mutations, especially during childhood and adolescence.

Magnetic resonance imaging of individuals with ATXPC revealed cerebellar atrophy in all patients and white matter hyperintensities in some. Cerebellar atrophy started during early childhood and was slowly progressive. The cerebellar atrophy is surprisingly pronounced compared to the generally mild balance impairment. Neuropathological examination of four individuals with ATXPC has been reported and brains showed pronounced loss of cerebellar Purkinje cells [13–15]. Granule cell loss was found in three patients but was less pronounced. There was depletion of white matter in the cerebellum in two individuals [13, 14] and in the hippocampus in one patient [15]. While the cerebellar atrophy can be readily explained by loss of Purkinje cells, cerebellar white matter and granule cells, these patients have been autopsied at ages when there commonly are no or no marked white matter hyperintensities in MRI. Thus, the exact neuropathological correlate of the white matter hyperintensities remains unknown, but we hypothesize that *SAMD9L* mutations may slow down the physiological myelination that physiologically occurs in childhood and adolescence.

## SAMD9

### Normal function of SAMD9

*SAMD9*, a highly conserved gene contigous to *SAMD9L*, shares a common gene structure to its paralogue and both genes encode proteins with 58% amino acid identity. Most mammals have both *Samd9* and *Samd9l*. However, mice have only *Samd9l*, and cows encode only *Samd9* suggesting that the genes partially complement each other’s functions [25]. *SAMD9*, like *SAMD9L*, is involved in control of cell proliferation and functions as a tumor suppressor in some cancers. Deleterious mutations in the *SAMD9* gene are known to cause normophosphatemic familial tumoral carcinosis (NFTC), a rare autosomal recessive disease. NFTC is characterized by abnormal inflammation of the skin and gingiva that is induced by excessive signaling via interferon pathways—in line with this it has been experimentally demonstrated that the function SAMD9 is tightly regulated by interferon-γ [26].

### SAMD9 mutations cause the MIRAGE syndrome

Recent work has identified mutations in *SAMD9* in children with myelodysplasia, infection, restriction of growth, adrenal hypoplasia, genital phenotypes, and enteropathy, named MIRAGE syndrome (OMIM #617053) (Figs. 1b and 2; Table 1) [27]. In the initial report the majority of the affected children died in early childhood, but two individuals with an expanded lifespan developed MDS with mononsomy 7, resulting in a loss of the mutant *SAMD9* allele. A recent study also reported that in seven MIRAGE children in whom parental DNA was available, the mutations were de novo and located in highly conserved residues, often affecting arginines (6 of 8) with half of all mutations clustering in a hotspot of codons 982 and 983 of *SAMD9* (Fig. 1b) [28]. The conclusion is therefore that *SAMD9* mutations underlying the MIRAGE syndrome are GOF and the negative effect on cellular growth puts a selection pressure on the cells to lose their mutated copy. In addition, this argues that the functions of these gene homologs are very similar. However, besides the similar hematologic phenotype, it remains elusive why the mutations in *SAMD9L* result in cerebellar atrophy while mutations in *SAMD9* are associated with complex multi-organ defects. We hypothesize that possible neurological manifestations in *SAMD9*-associated disease may have remained unnoticed because most patients died at a very young age when the nervous system is still immature and mild balance problems or visual problems would not have been evident.

Buonocore et al. describes eight MIRAGE patients with intrauterine growth restriction and severe testicular dysfunction leading to female external genitalia in six of the eight children, while the remaining two children had atypical external genitalia (Figs. 1b and 2) [28]. The majority of 19 de novo MIRAGE patients identified to date have had signs of bone marrow failure (various cytopenias) and infections in their medical history. Four children in total have had a longer life span and developed MDS with aberrations of chromosome 7. Strikingly, in the three non-MDS patients in which cytogenetics were performed, hematopoietic cells showed some form of aberration of chromosome 7, either monosomy 7 or 7q-. These three patients had an initial VAF around 50% but showed a lower VAF in a later sample indicating expansion of clones that that had lost their GOF *SAMD9* mutation due to monosomy 7 or 7q-. The reason that these patients did not develop MDS could be time related, since all died at 5–21 months of age. Similar to the observations made for GOF *SAMD9L* mutations in ATXPC, GOF *SAMD9* mutations were also reverted by other genetic mechanisms such as frame-shift or stop gain mutations in the blood of four individuals with MIRAGE syndrome (Fig. 3), including patients with non-manifest hematopoietic phenotype [28].

### Inherited SAMD9 mutation causing a milder phenotype

Schwartz et al. have recently identified a family with an inherited *SAMD9* mutation, in which three children developed MDS with monosomy 7 at an early age [29]. While the mother was completely unaffected, both by MDS and by genetic reversion, all three children displayed clonal somatic reversion in the blood by different mechanisms. One affected child had a CN-LOH of chromosome 7 and simultaneous monosomy 7, while the other two children had monosomy 7 as well as *in cis* mutations in *SAMD9*, arguing for neutralization of the germline GOF variant by both mechanisms.

It can only be speculated that the differences between de novo and familial SAMD9 mutations is the severity of the GOF. Weaker GOF variants are probably tolerated while more severe ones can only occur de novo due to embryonic lethality or is not fixated in the population due to infertility by the accompanying genital malformation. However, it is unclear in the reported family, why the mother did not develop any phenotype and did not show any genetic reversion. Unfortunately, RNA was not available for testing of uni- or biallelic expression of SAMD9 (J. Klco, Personal communication, May 2017). Importantly, this latest publication also provides additional evidence that different clones which have undergone genetic reversion by different mechanism, exist in the same patient and compete with each other resulting in the end in an outgrowth of the MDS clone with monosomy 7 [29].

## Diagnosis and follow-up

### Screening for SAMD9 and SAMD9L mutations

All patients with pediatric MDS with monosomy 7, del 7q and der(1;7) and probably all adult MDS patients with these chromosomal aberrations should be tested with gene panels not only covering *GATA2* associated disorders, but also including *SAMD9* and *SAMD9L* mutations on genetic material not obtained from blood cells. In our experience skin fibroblasts are the best source of germline DNA, whereas buccal swabs on some occasions have showed non representative skewing towards wt copies, likely due contamination of blood cells that have undergone reversion by CN-LOH [30]. Accompanying monocytopenia and low or absent NK cells shall even raise more suspicion about *GATA2* deficiency syndrome, ATXPC and MIRAGE syndromes, but this should not be a prerequisite for genetic testing because many patients with ATXPC and MIRAGE have “reverted” the GOF mutation resulting in a recovery of cell numbers in the PB.

With diagnosis of MIRAGE or ATXPC syndrome in one family member, a thorough family history should be taken and members of the family should, after consent, be offered genetic counseling and testing regarding the mutation identified in the family. Counseling should include the information that they may be mutation carriers, possibly at risk for MDS or cytopenias with potentially severe consequences, even if they have not experienced any symptoms previously. In ATXPC families, relatively subtle neurological signs may indicate carrier status. We consider testing family members because they may either be affected, at risk for developing MDS to our present knowledge, or since they might represent potential HSC donors for a family member with ATXPC syndrome. It should also be noted that some of the patients in the described families were suffering from clinical symptoms associated with low cell numbers of macrophages/monocytes as well as NK cells. So the recommendation for chest X-ray to exclude macrophage deficiency related alveolar proteinosis and screening vaccination for NK-cell deficiency related HPV infection is at the moment based on our clinical experience with a very limited patient cohort. However, we still think that the benefits of these screenings outweighs their risks and would recommend them until better data is available. In addition, for every carrier of pathogenic *SAMD9* or *SAMD9L* mutations without MDS we recommend frequent (every 6–12 months) follow-up physician visits and laboratory tests, as suggested in Table 2 [31].

**Table 2:** Recommendations for clinical care and surveillance of carriers of pathogenic mutations in SAMD9 or SAMD9L

At diagnosis	At follow up	Involvement of other specialists
• Clinical examination including basic neurological examination (test for balance and eye movement abnormalities) • Thorough family history • CBC with differential • Lymphocyte subpopulation analysis (to detect deficiencies in NK-cell and other populations) • Sequencing of SAMD9 or SAMD9L on genomic DNA obtained from a non-hematopoietic tissue • Sequencing of SAMD9 or SAMD9L on DNA from hematopoietic tissue to measure mutant VAF of the GOF mutation and to detect genetic reversion by somatic LOF mutations • Bone marrow biopsy with aspirate for karyotype and FISH for chromosome 7q as well as molecular analysis using targeted sequencing for genes mutated in myeloid malignancies to evaluate the risk for MDS transformation • SNP array on peripheral blood or bone marrow to estimate the extent of the CN-LOH of chromosome 7 or 7q • HLA typing • CXR (to look for alveolar proteinosis) • MRI of brain with T2/FLAIR imaging (for baseline assessment of cerebellar atrophy and white matter abnormalities; SAMD9L only)	• Clinical examination (hematology) every 6-12 months • CBC with differential every 6-12 months • CXR every third year (maybe every fifth year if the initial CXR was negative to minimize the exposure to radiation) • Gynecological examination once a year (but this is depending on the initial results, HPV vaccination status and on the identification of HPV strains) • Offer neurological and ophthalmological rehabilitation support as appropriate • Bone marrow aspirations for karyotype every other year or if any significant persistent change in CBC is detected • Targeted sequencing for genes mutated in myeloid malignancies to evaluate risk for MDS transformation every third year	• Neurologist (*SAMD9L* only) • Ophthalmologist (*SAMD9L* only) • Endocrinologist (*SAMD9* only) • Gynecologist (female patients) • Genetic Counseling • Reproductive medicine

*ATXPC* ataxia–pancytopenia syndrome, *CBC* complete blood count, *CN-LOH* copy number neutral loss of heterozygosity, *CXR* chest x-ray, *FISH* fluorescent in situ hybridization, *FLAIR* fluid-attenuated inversion recovery, *GOF* gain-of-function, *HLA* human leukocyte antigen, *LOF* loss of function, *MDS* myelodysplastic syndrome, *MRI* magnetic resonance imaging, *SNP*single-nucleotide polymorphism, *VAF* variant allele frequency.

### Treatment of MDS in SAMD9L and SAMD9 mutation carriers

Most difficult, regarding both syndromes, is to give recommendations on whom, when and how to perform hematopoietic stem cell transplantation (HSCT), because of a lack of evidence and extensive clinical experience. The 4-year ATXPC patient in the Swedish family developed severe neurological problems after allogeneic HSCT from a matched unrelated donor, although we still remain uncertain if there was a causal relationship [16]. Conditioning was performed according to the EWOG-MDS guidelines on HSCT in Childhood MDS and JMML, essentially using anti-thymocyte globulin, fludarabine and thiotepa for conditioning. Due to the fact that we were not aware of the definite diagnosis prior to the transplantation, a thorough neurological examination and a brain MRI were not performed during the pre-transplantation evaluation. However, MRI of the brain after the allogeneic HSCT showed pronounced cerebellar atrophy that correlated with onset of ataxia and nystagmus. This hypoplasia could be induced by a CMV infection the patient suffered during infancy, with prolonged problems due to his primary deficiency [32]. Moreover, no worsening of neurological symptoms in the transplanted boy in the Finnish family was observed [16]. However, it could be speculated that the conditioning triggered early onset of ATXPC related cerebellar atrophy, possibly by chemotherapy inducing an interferon response and that this in combination with the cellular damage imposed by these treatments will prevent cellular repair. Whether the same would apply to cellular damage induced by irradiation is of course unknown, but we would currently advise physicians to avoid higher doses of irradiation, especially to the brain, in patients with ATXPC syndrome.

With the currently available information it is impossible to draw any evidence-based conclusions, but we are worried that the only curative treatment for patients with ATXPC that develop MDS might aggravate the neurological symptoms and will severely affect their quality of life. Unfortunately, one can argue for both an early and a late time point for allogeneic HSCT. When transplanting before transformation to MDS, non-myeloablative regimens similar to the ones used for Fanconi’s anemia or *GATA2* deficiency syndrome can probably be used [33, 34], even though evidence for this assumption are missing at the moment. This idea is based on the observation that like in *GATA2* deficiency syndrome, the competitiveness of HSC is greatly reduced in ATXPC and MIRAGE syndrome and monocytes and NK cells are also reduced in number and possibly function. These effects reduce the risk for rejection and confer an advantage of the incoming HSCs in competition for niches in the bone marrow. Such reduced intensity conditioning will hopefully lessen any risk of developing cerebellar atrophy associated with allogeneic HSCT. However, once transformation to MDS with monosomy 7 has occurred, most transplant physicians would recommend a myeloablative conditioning, which at least in our patient possibly could have triggered cerebellar atrophy. We are reluctant to advice against this, but a careful risk-benefit analysis should of course be made, and of note should be that non-myeloablative conditioning regimens for HSCT after transformation are now in use for Fanconi anemia [35].

### Treatment of neurological symptoms in ATXPC

The neurological symptoms of ATXPC are generally mild and very slowly progressive, causing far less disability than other types of genetic cerebellar syndromes [36–38]. For the neurologist, considering the diagnosis and enquiring about any of the non-neurological disease features in the patient or family members is paramount. Once the diagnosis is verified, the patient can be reassured that the neurological disease usually will be slowly progressive and often remain mild to moderate until high age. Rehabilitation programs may be beneficial for those who experience balance problems. Awareness and specific measures for the less well-known form of vision impairment due to retinal dysfunction may reduce overall impairment. Access to dedicated neurology, ophthalmology and rehabilitation services should be provided. In the presently known families, there was complete or near-complete penetrance of neurological signs in *SAMD9L* mutation carriers. Thus, including neurological examination for, at the very least, balance and eye-movement abnormalities, in a hematology workup for cytopenia or MDS may faster lead to a suspicion of *SAMD9L-*related disease.

## Conclusions

GOF mutations in *SAMD9L*, located in the chromosome 7q, are responsible for the ATXPC syndrome. In all of the patients affected by myelodysplasia, aberrations of chromosome 7 resulted in a loss of the mutated *SAMD9L* allele during MDS transformation. Analysis of mutation carriers who went through a hematopoietic crisis at a young age, and who showed no symptoms at time of analysis, revealed CN-LOH of chromosome 7q not present at birth. Additionally, a LOF mutation in the same codon as the germline *SAMD9L* mutation occurred in one of these patients. De novo as well as familial germ-line mutations in the *SAMD9* gene have been discovered to cause the MIRAGE syndrome. The majority of MIRAGE patients died in infancy, but some developed a progressive loss of the mutated *SAMD9* allele through monosomy 7 or 7q-, and secondary LOF mutations. The latter eliminated the growth-restricting effects on the mutant SAMD9 protein in hematopoietic cells. However, a number of patients developed MDS with monosomy 7 or 7q- similar to what was observed in ATXPC. This illustrates that the elevated risk for MDS and AML in both syndromes are likely driven by monosomy 7 or 7q-, as a reversion mechanism gone awry, resulting in loss of the growth restricting imposed by the GOF mutation, but by secondary loss of genes in CDR1-3 on chromosome 7q contribute to leukemogenesis.
